# Using Diagnostic Radiological Imaging Modalities to Explore Neurological Dysfunction and Renal Failure in the Intersection of Hemophagocytic Lymphohistiocytosis, Macrophage Activation Syndrome, and Lupus

**DOI:** 10.7759/cureus.54005

**Published:** 2024-02-11

**Authors:** Juliana Cazzaniga, Kateryna Georgiyeva, Andrea Siguenza, Teresita Gonzalez, Pablo Ferraro

**Affiliations:** 1 Medicine, Florida International University, Herbert Wertheim College of Medicine, Miami, USA; 2 Internal Medicine, Memorial Healthcare System, Pembroke Pines, USA; 3 Neurology, Memorial Healthcare System, Pembroke Pines, USA; 4 Hematology and Oncology, Memorial Healthcare System, Hollywood, USA; 5 Hematology and Oncology, Memorial Healthcare System, Pembroke Pines, USA

**Keywords:** rheumatology, internal medicine, radiology, neurology, neuro-radiology

## Abstract

This comprehensive case report and literature review explore the intricate intersection of hemophagocytic lymphohistiocytosis (HLH), macrophage activation syndrome (MAS), and systemic lupus erythematosus (SLE) in a 39-year-old patient, emphasizing the challenging diagnostic and therapeutic landscape. The patient's journey includes neurological dysfunction, renal failure, and clinical complexities, showcasing the rarity of these overlapping conditions. The report explains the diagnostic process, clinical and laboratory findings, specialty consultations, and treatment decisions leading to the diagnosis of SLE with features of MAS overlapping with HLH. By offering insights into the latest research and clinical perspectives, this case report contributes to a deeper understanding of these disorders, aiming to guide clinicians in recognizing and managing such intricate cases effectively.

## Introduction

Macrophage activation syndrome (MAS) and Hemophagocytic lymphohistiocytosis (HLH) are rare and severe hyperinflammatory conditions that can have various clinical presentations on multiple organ systems, such as renal, bone marrow, liver, and spleen [1]. In some cases, these syndromes can manifest with atypical and alarming neurological symptoms such as seizures, cerebritis, and blindness [1]. This presentation is particularly challenging for both patients and healthcare providers, as it underscores the diverse and potentially life-threatening nature of these disorders [1]. This introduction provides a glimpse into the complexity of MAS and HLH when they involve the nervous system, emphasizing the critical need for early recognition and intervention. 

HLH, an exceptionally rare and often pediatric condition, carries an incidence rate of just 1 in 50,000 live births [2]. Its occurrence in adulthood, as observed in our patient, is exceedingly uncommon and introduces a distinctive diagnostic dilemma [2]. HLH is characterized by the uncontrolled activation of immune cells (histiocytes and lymphocytes), leading to a storm of inflammation that can affect various organs, including the liver, spleen, and bone marrow [2]. In the context of HLH, this uncontrolled immune response can become a life-threatening cascade due to organ failure, often necessitating urgent intervention [2].

MAS although distinct from HLH, shares a similar pathophysiological basis [3]. It is associated with the uncontrolled activation of macrophages and T lymphocytes, leading to an overwhelming cytokine storm and multi-organ involvement [3]. MAS frequently complicates underlying autoimmune diseases, such as SLE, further intertwining the clinical presentation [3].

Neurological involvement in the context of these disorders adds an additional layer of complexity [4]. Atypical neurological symptoms, such as seizures, cerebritis, blindness, and altered mental status, can serve as alarming indicators of a severe underlying condition [4]. Recognition and timely intervention are paramount in these cases, as delayed diagnosis and treatment can lead to devastating outcomes [4].

This introduction aims to provide a glimpse into the intricate nature of these conditions when they intersect, underscoring the critical need for early recognition and intervention. Our patient's journey through this complex medical landscape serves as a poignant example of the challenges that healthcare providers face when presented with such intricate scenarios.

The convergence of HLH, MAS, and SLE within a single patient is a clinical rarity that offers a unique opportunity for a comprehensive exploration of the diagnostic and therapeutic challenges. In the subsequent sections, we will meticulously dissect the case, offering insights into the diagnostic process, the myriad of clinical and laboratory findings, the consultations with various specialties, and the treatment decisions that ultimately led to a diagnosis of SLE with features of MAS, overlapping with HLH.

Furthermore, this case report and literature review aim to contribute to the broader understanding of these disorders by exploring the latest research and clinical perspectives. By delving into the intricacies of these intersecting conditions, we seek to provide valuable insights that may guide clinicians in recognizing and managing such intricate cases in the future, potentially improving diagnostic strategies and treatment approaches.

## Case presentation

A 39-year-old Jamaican female presented to the emergency room with a chief complaint of blurry vision persisting for two days. The patient reported a preceding day of headaches, fever, and dry cough associated with shortness of breath. She denied abdominal pain but mentioned nausea and a single episode of non-bloody, nonbilious emesis. Vital signs revealed blood pressure of 130/103, heart rate of 105, respiration rate of 20, oxygen saturation of 100%, and oral temperature of 38.0°C. Physical examination revealed fever, hepatosplenomegaly, lymphadenopathy, altered mental status, and skin malar rash. Laboratory workup revealed normocytic anemia, elevated serum creatinine, hyperbilirubinemia, and thrombocytopenia. The right upper quadrant ultrasound was unremarkable, but magnetic resonance venography (MRV) showed a venous aneurysm in the neck and showed outpouching, unclear if related to the confluence of venous structures versus a small venous aneurysm. A subsequent MRI of the orbits demonstrated abnormal signal intensity, mild edema, and enhancement of the optic nerve sheath complexes bilaterally. Differential diagnoses included idiopathic orbital inflammation, infectious or noninfectious systemic diseases, seronegative spondyloarthropathy, granulomatous disease, Behcet's, or lymphoproliferative disorders. Consultations with neurology, infectious disease, and ophthalmology were initiated. The patient was found to be RSV-positive and was diagnosed with posterior scleritis. Plasmapheresis and pulse steroid therapy methylprednisolone, intravenous 32 mg per day, were administered since an autoimmune process is more likely than infection.

Given the elevated ANA, RNP, anti-chromatin antibodies, and a recent episode of pericarditis, which suspicion of a connective tissue disease arose and subsequently was determined to be SLE. The patient underwent lumbar puncture and bone marrow biopsy, necessitating ICU placement due to acute respiratory failure and lethargy. Flow cytometry of cerebrospinal fluid (CSF) was predominantly composed of cellular debris and nonhematolymphoid elements with some lymphocytes and possibly some monocytes. The results concluded that no definite B-cell population was identified, and lymphocytes mostly consist of T-cells. Due to the limited specimen, T-cells could not be further characterized. Bone marrow from the right iliac crest resulted in mildly hypercellular marrow with trilineage hematopoiesis and complete maturation, dyserythropoietic changes, some atypical megakaryocytes, increased eosinophils, and no increase in blasts with rare hemophagocytic cells identified. Peripheral blood with mildly increased left shifted and immature granulocytes and increased schistocytes. Positive ANA/RNP, along with skin malar rash, posterior scleritis, and fever, led to the diagnosis of SLE. The bone marrow and CSF findings overlap with MAS and HLH. The subsequent management involved transfusion, plasmapheresis, pulse steroid therapy, and collaborative consultations with rheumatology and hematology-oncology. 

**Figure 1:**
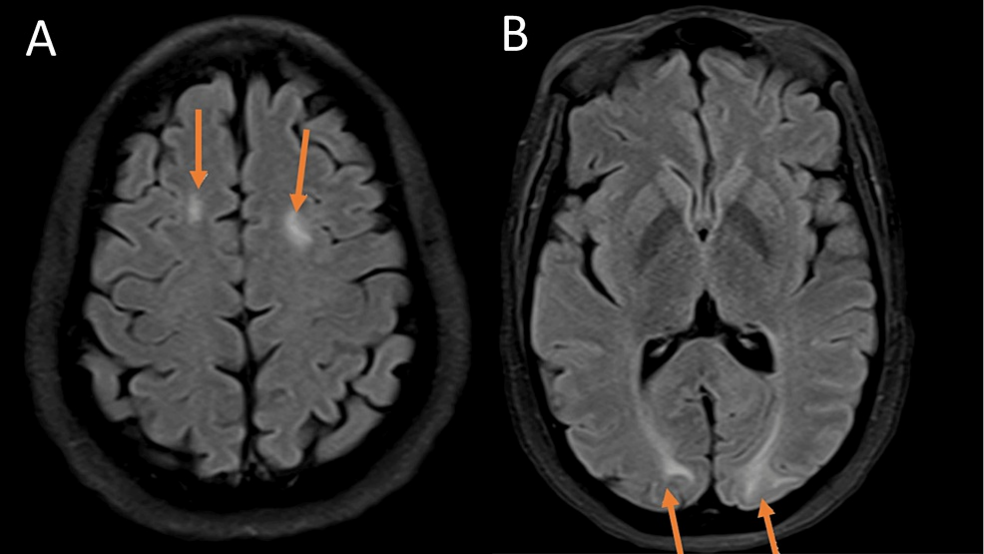
MRI without contrast Figure 1A demonstrates an axial view of the brain using MRI without contrast, demonstrating lupus cerebritis. There were new areas of increased T2/FLAIR signal in the bilateral occipital lobe subcortical white matter, the bilateral parietal periventricular white matter pointed by orange arrows and the bilateral frontal centrum semiovale. Additionally, there is a patchy high flair signal in the pons and a suspected small high T2 signal lesion in the right cerebellum. Figure 1B shows subtle petechial hemorrhages in the bilateral cerebellum and right pons, depicted by orange arrows.

**Figure 2:**
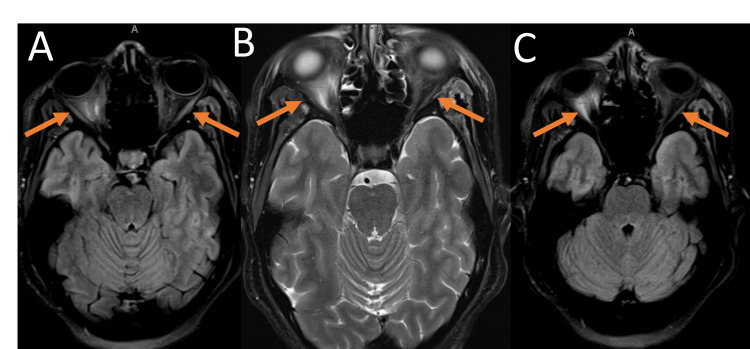
Multiple MRI without contrast highlighting bilateral orbits Figure 2A shows FLAIR MRI without contrast abnormal signal intensity in the intraconal orbits with mild edema and enhancement of the optic nerve sheath complexes bilaterally and enhancement of the posterior uveal scleral margin pointed by orange arrows. Figure 2B demonstrates T2-weighted MRI without contrast idiopathic orbital inflammation or a manifestation of an infectious or noninfectious systemic inflammatory disease shown by orange arrows. Figure 2C shows FLAIR MRI, which also highlights the orbital inflammatory changes in orange arrows.

Magnetic resonance imaging (MRI) of the brain without contrast exhibiting lupus cerebritis (Figure 1-2). Figure 1 shows there are areas of increased T2/FLAIR signal in the bilateral occipital lobe subcortical white matter, the bilateral parietal periventricular white matter, and the bilateral frontal centrum semiovale. Additionally, there is a patchy high flair signal in the pons and a suspected small high T2 signal lesion in the right cerebellum. There are also subtle petechial hemorrhages in the bilateral cerebellum and right pons. Although this is nonspecific, it is suggestive of Posterior Reversible Encephalopathy Syndrome (PRES), which can be seen in the setting of lupus [5]. Figure 2 demonstrates abnormal signal intensity in the intraconal orbits with mild edema and enhancement of the optic nerve sheath complexes bilaterally and the posterior uveal scleral margin enhancement. Differential diagnoses include seronegative spondyloarthropathy, granulomatous disease, Behcet's disease, or lymphoproliferative disorder versus an infectious ocular etiology, including posterior scleritis/uveitis or endophthalmitis.

Table 1 displays laboratory results, including the reference range for each parameter. The chemical profile provides a comprehensive snapshot of the individual's physiological condition. Key observations include a slightly high glucose level, indicated by an elevated glucose level. The BUN level suggests impaired kidney function. Sodium exhibits a decrease, while chloride and bicarbonate remain within the normal range. The anion gap is slightly below the typical range. Corrected calcium is 9.2 mg/dl (within range of normal calcium levels). Serum creatinine is notably high, signaling potential kidney filtration impairment. Serum albumin is a known negative acute phase reactant and can be low when there is an inflammation or infection, in addition to liver disease and other reasons. Liver function markers ALT and AST show elevated levels and total bilirubin is raised, hinting at a potential hepatic concern. Phosphorus levels are elevated, and reduced TIBC suggests a possible iron deficiency. These findings in Table 1 collectively point to a complex interplay of metabolic and organ-specific imbalances, warranting further clinical evaluation.

**Table 1:** Laboratory results of the chemical profile, including reference ranges This demonstrates the blood work results, depicting the up arrow (↑) for increased result, and down arrow (↓) for decreased result. Interleukin-2 is a sensitive diagnostic test in adult HLH. HLH: Hemophagocytic Lymphohistiocytosis

Chemical Profile (units)	Values	Reference Range
Glucose (mg/dL)	100 ↑	70-99
Blood Urea Nitrogen (BUN) (mg/dL)	19 ↑	2.1 - 8.5
Sodium (mmol/L)	133 ↓	135 - 145
Potassium (mmol/L)	4	3.6 - 5.2
Chloride (mmol/L)	100	96 - 106
Bicarbonate (mEq/L)	29	23 - 29
Anion gap (mEq/L)	4 ↓	5-12
Calcium (mg/dL)	8.1 ↓	8.5 - 10.5
Creatinine (mg/dL)	2.06 ↑	0.74 - 1.35
Total protein (g/dL)	6.4	6.0 - 8.3
Albumin (g/dL)	2.6 ↓	3.4 - 5.4
Alkaline phosphatase (IU/L)	61	44 - 147
Alanine transaminase (ALT) (U/L)	82 ↑	7 - 56
Aspartate transferase (AST) (U/L)	164 ↑	8 - 33
Bilirubin total (mg/dL)	1.7 ↑	0.1 - 1.2
Magnesium (mmol/L)	1.7	1.3 - 2.1
Phosphorus (mmol/L)	5.0 ↑	2.8 - 4.5
Iron (μmol/L)	87	60 - 170
Total iron-binding capacity (TIBC) (mcg/dL)	226 ↓	240-450
lactate dehydrogenase (U/L)	793 ↑	140 - 280
ferritin (ng/mL)	>10,000 ↑	12 - 150
triglycerides (mg/dl)	373 ↑	<150
total cholesterol (mg/dL)	147	<200
Low-density lipoprotein (mg/dL)	51	<100
High-density lipoprotein (mg/dl)	55	>50
Interleukin-2 (pg/ml)	6361 ↑	0-5

Table 2 demonstrates complete blood count test results of the patient depicting thrombocytopenia and decreased red blood cells congruent with the etiology of MAS and HLH. 

**Table 2:** Complete blood count test results

Complete Blood Count (units)	Values	Reference Range
White blood cells (k/ul)	15.7 ↑	4.5-11
Red blood cells (k/ul)	3.14 ↓	3.5-5.5
hemoglobin (g/dl)	8.6 ↓	12-16
hematocrit (%)	26.4 ↓	41-50
mean corpuscular volume (fl)	84.1	80-100
Mean corpuscular hemoglobin concentration (g/dI)	32.6	32-36
red cell distribution width (%)	22.6 ↑	11.5–15.4
platelets (k/ul)	24 ↓	150-450

In the corresponding peripheral blood smear, the white blood cells are mildly increased with predominant left-shifted granulocytes, including a few immature granulocytes. No blasts were seen. There were rare atypical lymphocytes and rare hyposegmented neutrophils. A manual differential cell count revealed 47% segmented neutrophils, 20% band neutrophils, 22% lymphocytes, 2% monocytes, 4% eosinophils, and 5% myelocytes. The red blood cells showed anisopoikilocytosis with increased schistocytes, rare target cells, few elliptocytes, and few polychromatophilic cells. There were a few nucleated red blood cells. The platelets were decreased, and some were large in size. Rare giant platelets were seen. A concurrent flow cytometric study (F23-03183) revealed less than 1% myeloid blasts. Phenotypic eosinophils comprised approximately 33% of nonerythroid cells. No monoclonal B cells or phenotypically aberrant T cells were identified. A small subset of monocytic cells showed aberrant expression of CD56.

Plasma cells comprised approximately 1% of cells and showed polyclonal cytoplasmic light chain expression, with a kappa to lambda ratio of approximately 2.6:1. The marrow was mildly hypercellular, showing trilineage hematopoiesis with complete maturation, dyserythropoietic, changes, some atypical megakaryocytes, increased eosinophils, and no increase in blasts. There are rare hemophagocytic cells and some bacterial-like structures on aspirate smears. There is mild leukocytosis in peripheral blood with left shifted and immature granulocytes and increased schistocytes. The dyserythropoietic changes and atypical megakaryocytes are nonspecific and could be reactive, such as "stress dyserythropoietic" (with the described history of hemolytic anemia and autoimmune disorder). However, correlation with concurrent cytogenetic and molecular studies is required to exclude any underlying hematopoietic neoplasm/myelodysplastic syndrome. The increased eosinophils were nonspecific and could have been reactive, as seen in parasitic infections, allergies, pulmonary disease, skin diseases, collagen vascular diseases, and Kimura's disease, among others. However, a neoplastic process with increased eosinophils was in the differential diagnosis, and correlation with cytogenetic studies was recommended.

Her chromosome analysis was abnormal: 46, XX, t(2:4) (g37; q12) [21/46, XX (18)]. The identified translocation in this specimen lacks specificity for a particular hematopoietic malignancy. The clinical significance of detecting only two abnormal cells remains unclear, underscoring the need for correlation with hematopathology and subsequent bone marrow studies, including cytogenetic analysis. This comprehensive approach is crucial to offer a thorough interpretation of the findings and to rule out the presence of a significant disease-associated clone. Notably, concurrent Fluorescence In Situ Hybridization (FISH) studies were negative for PDGERA gene rearrangement located at 4q12, providing additional context to the genetic landscape observed in this specimen. The integration of these results emphasizes the importance of a multifaceted diagnostic strategy for a comprehensive understanding of the specimen's molecular profile.

The assessment reveals that the factor H was within the normal range, shedding light on a notable aspect of this complex case. The diagnostic journey was further enriched by negative results for Shiga toxin and factor H, making it less likely that the patient's condition involves complement-mediated Thrombotic Thrombocytopenic Purpura (TTP) or Hemolytic Uremic Syndrome (HUS). The patient's intricate clinical presentation involves a myriad of symptoms, sparking a thorough diagnostic investigation. Notably, the initial assessment revolves around MAHA, characterized by Coombs-negative hemolysis, thrombocytopenia, and the potential presence of MAS. The differential diagnosis encompasses considerations for HLH, TTP (though less likely), and MAS linked to SLE or autoimmune-related hemolysis associated with connective tissue disease. The presence of eosinophilia, positive markers such as ANA, RNP/SM, SSA, and a positive RSV test further complicates the diagnostic landscape. The patient's initial symptoms included visual disturbances, fever, a platelet count of 50, and signs of hemolysis, including mildly elevated indirect bilirubin, lactate dehydrogenase (LDH) at 2172, 1+ schistocytes, and a reticulocyte count of 4.3%. Iron panel results suggested an anemia of chronic disease, with ferritin exceeding 10,000, while folate and vitamin B12 levels remained within normal limits. 

Laboratory findings indicated a positive RSV test, Coombs negativity, and notable results from a peripheral blood examination, including a left shift of myeloid cells and increased eosinophils (20% of cells). Further tests revealed no monoclonal B-cell population or phenotypically abnormal B-cell population with Epstein-Barr virus (EBV) negativity. Abdominal imaging displayed no hepatosplenomegaly.

Serum markers such as elevated CD25 (>6000), ferritin (>10,000), triacylglycerol (TAG) at 373, bicytopenias, fevers, and fibrinogen <250, in conjunction with abnormal liver function tests, posed a diagnostic challenge. Repeated ferritin levels showed improvement at 1870, while CXCL9 was elevated at 14176. CD107/NK remained normal, and a subsequent bone marrow biopsy on October 5, 2023, revealed rare hemophagocytic cells.

The management plan involves the patient meeting 5/5 criteria for HLH (given the elevated CXCL9) and a bone marrow positive for hemophagocytic cells. However, due to abnormal liver function and hepatomegaly, MAS associated with SLE is deemed likely, presenting an overlap with HLH. TTP is considered less likely, given the significant presence of schistocytes, renal and central nervous system involvement, and MAHA, with ADAMTS13 levels above 10%. The nuanced intricacies of this case necessitate a meticulous, multidisciplinary approach to unravel the diagnostic puzzle and formulate an effective treatment strategy.

**Figure 3:**
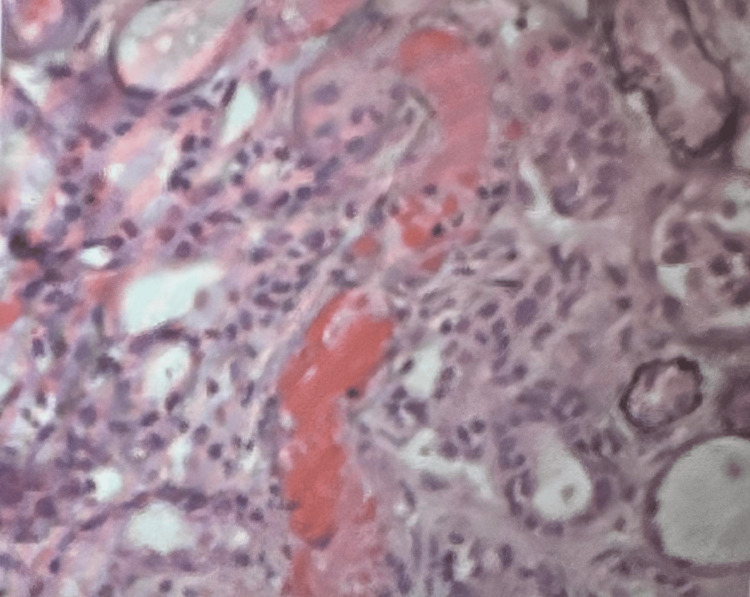
Kidney biopsy

Additional laboratory investigations were initiated in response to eosinophilia, a feature associated with autoimmune/connective tissue disorders and specific hematologic conditions. Tests including MDS Fish, FGFR1, PDGERa/b, and NGS myeloid were performed and returned negative for her eosinophilia. The MAHA presentation remains partially explained, prompting continued scrutiny of factor H levels to assess for complement-induced Thrombotic Microangiopathy (TP), with results pending. Evaluation for autoimmune Heparin-Induced Thrombocytopenia (HIT) through PF4 testing yielded negative results, mitigating concerns about drug-induced eosinophilia. Clinically improved, the patient currently does not require etoposide for HLH, allowing the continuation of steroids to address underlying SLE. Plasma exchange remains deferred, and ongoing steroid therapy covers potential treatment for both TTP, HLH and any rheumatologic conditions. Rheumatology initiated SLE treatment with CellCept, and cardiac assessment revealed a negative echo for valvular pathology, eliminating its contribution to red blood cell shearing. Figure 3 demonstrates a left native kidney biopsy with diffuse thrombotic microangiopathy and severe acute tubular injury/necrosis. This suggests a vascular component contributing to renal impairment.

## Discussion

The treatment approach involved a combination of plasmapheresis, pulse steroids, and disease-modifying antirheumatic drugs (DMARDs), reflecting the need for aggressive immunosuppression to control the hyperinflammatory response [6]. Collaborative efforts among specialists were crucial in guiding treatment decisions, especially when faced with conflicting clinical features [6]. The significance of considering alternative diagnoses, such as TTP, given the patient's clinical presentation [6]. Thorough evaluation, including hematological parameters and neuroimaging, was pivotal in ruling out other potential causes [6].

HLH, MAS, and SLE convergence within a single patient presents a diagnostic and therapeutic challenge [7]. The complexity arises from the rarity of these conditions individually, making their coexistence exceptionally uncommon [4,7]. This discussion explores key aspects of the case, including the diagnostic process, clinical manifestations, and treatment strategies [7].

The patient's initial presentation with neurological symptoms, including blurry vision, seizures, and altered mental status, underscored the severity of the conditions and highlighted the need for early recognition. Such atypical neurological involvement in the context of HLH, MAS, and SLE adds a layer of complexity, necessitating a multidisciplinary approach involving radiology, neurology, hematology-oncology, rheumatology, and ophthalmology [8]. The patient's diagnostic journey revealed overlapping features of these syndromes, making it challenging to distinguish one from another. The elevated ANA, RF, and positive RNP/anti-chromatin antibodies suggested a connective tissue disease, eventually leading to the diagnosis of SLE with features of MAS overlapping with HLH [8]. This intricate diagnostic process emphasizes once again the importance of considering a broad differential and collaborating across various medical specialties. Renal involvement, as evidenced by acute kidney injury (AKI), further complicated the clinical picture [9]. The patient's underlying rheumatologic disease and the presence of RSV as a triggering factor added layers of complexity, requiring careful management to address both the infectious and autoimmune components [9].

The peripheral blood analysis reveals an increase in schistocytes, a nonspecific finding but, when coupled with thrombocytopenia, raises concerns about potential thrombotic microangiopathy. Additionally, the identification of rare hemophagocytic cells, while nonspecific, prompts consideration for HLH and/or MAS. HLH can manifest as either familial or acquired, often secondary to infectious, autoimmune, or neoplastic conditions. The diagnostic landscape is further complicated by the association of autoimmune diseases, particularly rheumatologic disorders, with an HLH-like syndrome, collectively termed macrophage activation syndromes. This intricate interplay of hematological findings underscores the need for a meticulous diagnostic approach to delineate the underlying factors contributing to the patient's complex clinical presentation.

The intersection of HLH, MAS, and SLE in this patient exemplifies the complexity of autoimmune and hyperinflammatory disorders [10]. The challenges in diagnosis and management underscore the importance of a systematic and collaborative approach involving various medical specialties [11]. This case contributes valuable insights to understanding these rare conditions, emphasizing the need for continued research and sharing of clinical experiences to improve the management of such intricate cases in the future [12,13].

Around 35% of MAS patients experienced CNS dysfunction, including seizures and mental status alterations, often accompanied by irritability, lethargy, comas, and headaches [13]. These CNS manifestations were more pronounced in MAS compared to active SJIA but weren't considered early MAS onset indicators [13]. Hemorrhagic manifestations, from easy bruising to severe bleeding, occurred in 20% of MAS patients. Severe MAS cases led to multiple organ failures and fatal outcomes [13]. Diagnosing MAS early was challenging as clinical features were not immediately apparent; they became more evident as MAS progressed [13]. Relying solely on clinical presentation posed limitations in achieving early MAS diagnosis [13].

HLH diagnosis can be established through fulfillment of Criterion 1 or 2. Criterion 1 involves a molecular diagnosis consistent with HLH [14]. For Criterion 2, five of the eight specified criteria must be met, including fever, splenomegaly, cytopenias affecting at least two of three lineages in peripheral blood, hypertriglyceridemia and/or hypofibrinogenemia, hemophagocytosis in bone marrow or spleen or lymph nodes, low or no NK cell activity, ferritin ≥500 μg/L, and sCD25 (soluble IL-2 receptor) ≥2400 U/mL [14]. Further exploration is recommended in cases where hemophagocytic activity is not initially proven [14]. Hyperferritinemia, often exceeding 7000 to 10,000 µg/L in adults, is a key indicator, particularly when accompanied by other clinical features such as hyperbilirubinemia, hepatomegaly, transaminitis, and elevated lactate dehydrogenase and d-dimer levels [14]. While ferritin levels >10,000 µg/L are highly sensitive and specific in children, additional criteria are essential for a diagnosis [14]. Diagnostic confirmation in adults requires an integrative approach considering various clinical features, as the HLH-2004 criteria, designed for children, are not formally validated for adults [14]. Other supportive diagnostic features include spinal fluid pleocytosis, elevated spinal fluid protein, histological liver changes, and additional laboratory findings consistent with HLH, aiding in distinguishing it from other conditions and monitoring treatment response [14].

Thrombotic Microangiopathy (TMA) in SLE can manifest either as renal-limited or present with systemic features [14]. It is crucial to differentiate it from secondary TTP by assessing ADAMTS13 activity and antibodies due to their therapeutic implications [14]. Contrary to expectations, elevated anti-DNA antibody titers and hypocomplementemia do not reliably predict renal TMA in lupus nephritis [14]. TMA is a relatively rare occurrence (3%-9%) in SLE and significantly contributes to poor renal outcomes, independent of the presence of antiphospholipid antibodies [14]. In a study involving 148 lupus nephritis biopsies, 24% presented with TMA, with 80% being isolated cases and 20% associated with other conditions like TTP, HUS, malignant hypertension, and scleroderma [14]. Patients with TMA exhibited worse kidney outcomes, particularly when C4d staining was positive and serum FH levels were low [14]. The complement system in SLE has a dual role-protective in clearing apoptotic cells and immune complexes, yet pathogenic in amplifying inflammatory responses [14]. Genetic analysis suggests that an intact classical pathway is crucial for preventing lupus nephritis, and defects in alternative pathway genes (FH, factor I, large deletions in CFHR1-CFHR3, possibly through anti-FH antibodies) have been associated with SLE and lupus nephritis [14]. Despite evidence of genetic abnormalities in complement pathways contributing to lupus nephritis, the role of genetic variants in patients with TMA in lupus remains less explored [14]. In cases of persistent TMA despite lupus nephritis treatment, a study involving 43 patients with SLE/APS demonstrated positive outcomes with eculizumab, indicating the potential involvement of complement pathways [14].

TMA is characterized as a pattern of injury rather than a specific pathological diagnosis. It encompasses a broad spectrum of potential underlying etiologies, including HUS (associated with Shiga toxin or complement dysregulation), TTP, Antiphospholipid Antibody Syndrome, scleroderma renal crisis, SLE, drug or radiation-induced diseases, and malignant hypertension. Notably, TMA can manifest in patients clinically diagnosed with systemic lupus erythematosus, regardless of the presence of class III or IV lesions indicative of lupus nephritis. In the current case, although lupus nephritis is not evident, the presentation includes thrombotic microangiopathy, a phenomenon observed in individuals exhibiting clinical symptoms associated with systemic lupus erythematosus. This underscores the complexity of the patient's condition, necessitating a thorough exploration of potential contributory factors within the intricate web of autoimmune and vascular dynamics.

In this multifaceted clinical scenario, the presence of RSV introduces a viral element, suggesting a potential trigger or contributor to the patient's overall health challenges. The concurrent occurrence of HLH and MAS underscores the hyperinflammatory state, with activated macrophages playing a pivotal role in both syndromes. The connection with SLE adds an autoimmune dimension, as SLE is known for its wide-ranging impact on various organ systems. TMA further complicates the vascular aspect, potentially contributing to microvascular thrombosis and end-organ damage. Identifying an abnormal female karyotype brings a chromosomal anomaly into the narrative, posing additional questions about its role in shaping the patient's clinical presentation. Unraveling the intricate connections among these elements is imperative for a holistic understanding, enabling healthcare professionals to tailor interventions that address the underlying complexities of this unique case.

## Conclusions

Imaging modalities played a pivotal role in unraveling the complexity of the presented case involving the convergence of HLH, MAS, and SLE. The utilization of CT arteriogram and MRI of the brain and orbits proved crucial in identifying abnormal signal intensities, enhancing the optic nerve sheath, and revealing venous anomalies. These findings provided insights into the neurological manifestations and guided subsequent consultations with specialists such as diagnostic radiology, neurology, neuro-interventional radiology, and ophthalmology. The detailed imaging allowed for a thorough exploration of potential diagnoses, differentiating between rheumatologic, infectious, and idiopathic etiologies. Additionally, the identification of a venous aneurysm through imaging highlighted the systemic involvement of these conditions. The imaging results significantly informed the diagnostic process, particularly the consideration of TTP, reinforcing the critical role of radiological assessments in guiding clinical decision-making and ultimately contributing to the comprehensive understanding of this intricate case.
